# Modulation of chromatin remodeling proteins SMYD1 and SMARCD1 promotes contractile function of human pluripotent stem cell-derived ventricular cardiomyocyte in 3D-engineered cardiac tissues

**DOI:** 10.1038/s41598-019-42953-w

**Published:** 2019-05-16

**Authors:** Maggie Zi-Ying Chow, Stephanie N. Sadrian, Wendy Keung, Lin Geng, Lihuan Ren, Chi-Wing Kong, Andy On-Tik Wong, Jean-Sebastien Hulot, Christopher S. Chen, Kevin D. Costa, Roger J. Hajjar, Ronald A. Li

**Affiliations:** 1 Stem Cell and Regenerative Medicine Consortium, The University of Hong Kong, Pok Fu Lam, Hong Kong; 2 School of Biomedical Sciences, The University of Hong Kong, Pok Fu Lam, Hong Kong; 3 Ming Wai Lau Centre for Reparative Medicine, Karolinska Institutet, Shatin, Hong Kong; 4 Cardiovascular Research Center, Icahn School of Medicine at Mount Sinai, New York, New York, USA; 5 Dr. Li Dak-Sum Research Centre, The University of Hong Kong, Pok Fu Lam, Hong Kong; 6 Sorbonne Universités, UPMC Univ Paris 06, Institute of Cardiometabolism and Nutrition (ICAN), Pitié-Salpêtrière Hospital, F-75013 Paris, France; 7 Department of Biomedical Engineering, Boston University, Boston, Massachusetts USA; 8 The Wyss Institute for Biologically Inspired Engineering, Harvard University, Boston, USA

**Keywords:** Epigenetics, Embryonic stem cells, Heart stem cells

## Abstract

Human embryonic stem cells (hESCs) and induced pluripotent stem cells (iPSCs) have the ability of differentiating into functional cardiomyocytes (CMs) for cell replacement therapy, tissue engineering, drug discovery and toxicity screening. From a scale-free, co-expression network analysis of transcriptomic data that distinguished gene expression profiles of undifferentiated hESC, hESC-, fetal- and adult-ventricular(**V)** CM, two candidate chromatin remodeling proteins, SMYD1 and SMARCD1 were found to be differentially expressed. Using lentiviral transduction, SMYD1 and SMARCD1 were over-expressed and suppressed, respectively, in single hESC-**V**CMs as well as the 3D constructs Cardiac Micro Tissues (CMT) and Tissue Strips (CTS) to mirror the endogenous patterns, followed by dissection of their roles in controlling cardiac gene expression, contractility, Ca^2+^-handling, electrophysiological functions and *in vitro* maturation. Interestingly, compared to independent single transductions, simultaneous SMYD1 overexpression and SMARCD1 suppression in hESC-**V**CMs synergistically interacted to increase the contractile forces of CMTs and CTSs with up-regulated transcripts for cardiac contractile, Ca^2+^-handing, and ion channel proteins. Certain effects that were not detected at the single-cell level could be unleashed under 3D environments. The two chromatin remodelers SMYD1 and SMARCD1 play distinct roles in cardiac development and maturation, consistent with the notion that epigenetic priming requires triggering signals such as 3D environmental cues for pro-maturation effects.

## Introduction

Human adult cardiomyocytes (CMs) are terminally differentiated and non-regenerative. Using *in vitro* differentiation methods, ventricular (**V**) CMs derived from human embryonic stem cells (hESCs) or induced pluripotent stem cells (iPSCs) are a valuable source of genuine heart **V**CMs for cell-based transplantation therapies and as models for drug screening and discovery (reviewed in^1,2^). However, an accepted major impedance has been the immaturity of hESC/iPSC-**V**CMs in such properties as contractility, calcium handling and electrophysiology. We previously reported that the epigenetic state of hESC-**V**CMs is dynamic and primed to promote growth and developmental maturation, but that specific environmental stimuli with chromatin remodeling will be required to synergistically trigger maturation to a more adult-like phenotype^1,3^. As a key mode of transcriptional regulation, however, chromatin remodeling has not been extensively investigated in cardiac differentiation and maturation of hESC/iPSC-**V**CMs. Indeed, distinct chromatin and gene expression patterns are known to associate with lineage and cell fate decisions^4,5^. In the present study, a number of chromatin remodeling proteins that are differentially expressed in hESC-**V**CMs compared with undifferentiated hESC, fetal- and adult-**V**CMs have been identified. Using a multi-scale bioinformatics approach, two chromatin remodeling proteins, SMYD1 and SMARCD1, were chosen for further investigating their roles in the contractile and electrophysiological maturation of hESC-**V**CMs by comparing the functional consequences of engineering their expression levels in 2D and 3D models.

## Results

### Predicting novel regulatory motifs using co-expression network analysis

Previously, our laboratory has compared the gene expression profiles of undifferentiated hESC, hESC-**V**CM, fetal-**V**CM and adult-**V**CM in a microarray analysis^6^. The dataset consists of expression data for 13,371 array probes that map to protein-coding genes and are expressed in at least one cell type. To gain a deeper understanding of co-regulatory elements in the dataset, the WGCNA package in R was used to identify scale-free networks of co-expressed genes. WGCNA is a reductionist method of analyzing large datasets by grouping genes into modules or hierarchical groups, based on co-expression. Specifically, genes are grouped by connectivity, which is the sum of pairwise correlations between a given gene and all other genes within an array, raised to a power β. The power transformation increases the relative importance of strong correlations and diminishes the relative importance of weak correlations, and hence provides a robust and effective way in identifying biologically relevant gene clusters, without a priori knowledge of gene function, in a variety of biological contexts^7–9^. By converging multiple analyses of a single data set, including differential expression analysis and network-based co-expression analysis at multiple developmental stages, a more accurate picture of ventricular cardiomyocyte development and maturation can be captured.

**Figure 1:**
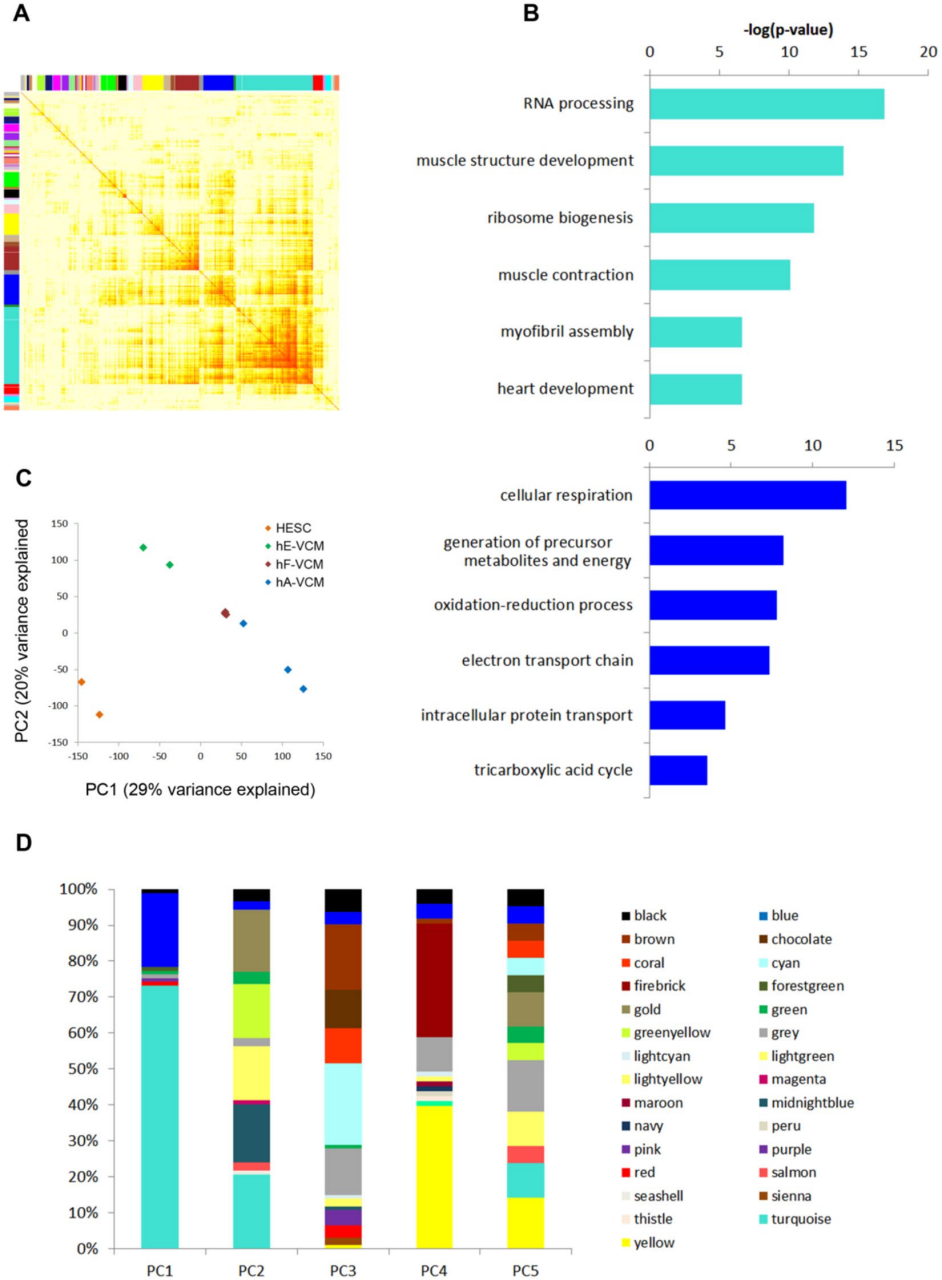
Microarray analysis of hESC, hESC-VCM, fetal-VCM and adult-VCM. (**A**) Weighted gene co-expression network analysis (WGCNA) has grouped co-expressed genes into color-coded modules. The x and y axes represent all protein-coding genes in our analysis and their pairwise correlations are represented as a point of color. Color of each pairwise gene comparison indicates Pearson correlation with red representing a correlation of 1. **(B)** The top biological functional groups for genes belonging to the turquoise and blue modules. **(C)** The first two principal components account for ~50% of the variance observed between samples. **(D)** Module assignment of the genes making up the top 100 ranked genes by weight in principal components 1–5 from principal component analysis (PCA).

The results were visualized as a topography map (Fig. 1A), in which the X and Y axes each correspond to the entire dataset hierarchically clustered by the default dissimilarity measure within the WGCNA package^10–13^. Genes that were expressed with greater correlation and topological similarity cluster more closely together into color-coded modules. Enriched functional groups were identified for each module using Gene Ontology data.

The largest module, termed “turquoise”, containing 2659 unique protein-coding genes, was enriched for gene sets involved in RNA processing, cardiac development, and muscle contraction. The “blue” module, containing 1137 unique genes, was enriched for gene sets involved in cellular respiration, redox reactions, and mitochondrial functions. Together, the turquoise and blue modules were enriched for biological functions found to be depleted in hESC-**V**CMs (Fig. 1B). Principal component analysis (PCA) was performed using the princomp function with default settings in Matlab (Fig. 1C). By ranking the significance of contribution of each gene by weight, the turquoise and blue modules were over-represented in the top ranked 100 genes contributing to the first principal component (Fig. 1D). Among these 100 genes, 71 genes belong to the turquoise module, which were three times more than expected by random chance (x^2^ = 121.12, P = 3.62E-28); while 20 genes belonging to the blue module were twice more than expected (x^2^ = 11.11, P = 8.58E-4). Together, these results suggested that the turquoise module is the most relevant network containing co-expressed genes necessary for a mature **V**CM phenotype.

**Figure 2:**
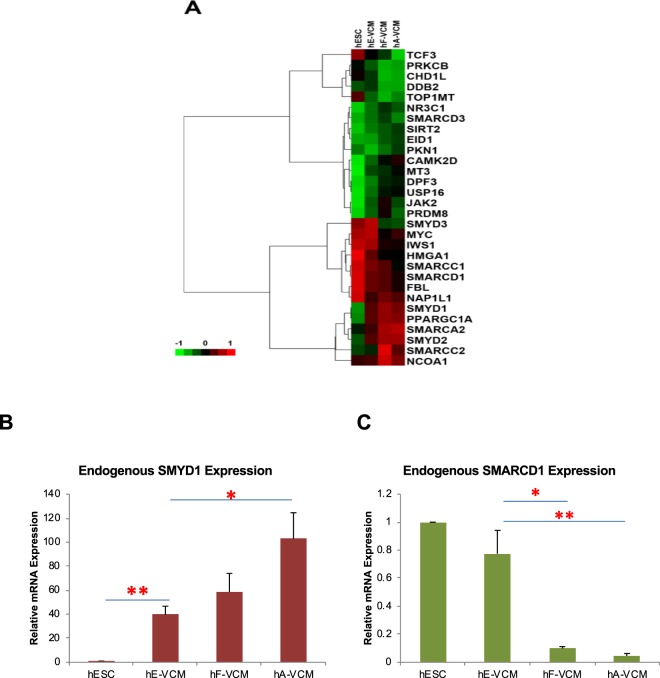
Expression of chromatin remodelling proteins in hESC, hESC-VCM, fetal-VCM and adult-VCM. (**A**) A heat map that summarizes expression of all genes involved in chromatin remodeling (GO:0016568) assigned into the turquoise module. The endogenous expression of **(B)** *SMYD1* and **(C)** *SMARCD1* in undifferentiated hESC, hESC-**V**CM, fetal-**V**CM and adult-**V**CM is validated by quantitative real-time PCR. S.E.M. of three independent experiments are presented. *p < 0.05; **p < 0.01 (Student’s t-test).

To identify novel transcriptional regulatory motifs involved in cardiac maturation, the turquoise module gene list was queried to a set of 3410 genes implicated in transcriptional regulation – sourced from InterPro, TRANSFAC, DBD, and GO databases^14^. Overall, 544 transcriptional regulators were found within the turquoise module. Interestingly, of these, 117 (22%) genes have been previously associated with cardiac or muscle development according to the Gene Ontology database. Of the transcriptional regulators within the turquoise module, 97 genes were predicted to function as chromatin remodelers, and of these, 30 genes were significantly differentially expressed in hESC-**V**CMs compared with either fetal- or adult-**V**CMs (Fig. 2A). Based upon their co-expression within the cardiac-relevant turquoise co-expression module, and differential expression in hESC-**V**CMs, the data suggested that the genes identified could play a role in ventricular specification or maturation.

Of the 30 candidate genes, 13 genes have an expression level above the threshold (between 0 to +1) in hESC-**V**CMs, which include members of the SMYD family (SMYD1, SMYD2, and SMYD3) and the SWI/SNF-related complex (SMARCA2, SMARCC1, SMARCC2, and SMARCD1). SMYD1 (SET- and MYND-domain) proteins are key regulators in skeletal and cardiac muscle development and function^15^. The SET (Su(var)3–9, Enhancer of Zeste, Trithorax) domain is responsible for the transfer of methyl groups to specific lysine residues of histones; the MYND (myeloid, Nervy, DEAF-1) domain is a zinc finger domain with potential to elicit protein-protein interactions. Among the SMYD family members, SMYD1 has been shown to play the most critical role in heart development and cardiomyogenesis. SMYD1 is expressed in the cardiac primordium and heart in developing embryos and deletion of Smyd1 is embryonically lethal in mice^16^. SMYD2 is involved in skeletal muscle development and myofibril organization^17^. SMYD3 regulates expression of myogenic markers and is involved in skeletal muscle atrophy^18^. Multiple subunits of the SWI/SNF chromatin remodeling complex have been identified to physically and functionally interact with cardiogenic transcription factors important in cardiovascular development and diseases (reviewed in^19^). By contrast, SMARCD1 (SWI/SNF related, matrix associated, actin dependent regulator of chromatin, subfamily d, member 1) is homologous to Baf60a in mouse. SMARCC1 and SMARCC2 are present in BRG1-SWI/SNF complex, which plays a repressive role at the enhancers of lineage-specific genes in hESCs^20^. SMARCD1 is a key component in cardiac progenitors and interacts with Tbx1 to regulate the transcription of Wnt5a in mice^21^. As shown in Fig. 2, *SMYD1* expression was abundant in fetal and adult, but not hESC-**V**CMs. On the other hand, *SMARCD1* displayed the opposite pattern of expression – it was robustly expressed in undifferentiated hESC and declined developmentally in hESC-**V**CMs, fetal-**V**CM and adult-**V**CMs. These microarray-based data were further validated using quantitative real-time PCR (Fig. 2B,C). Given the expression patterns SMYD1 and SMARCD1 as well as their known intriguing roles in the heart, the two chromatin remodelers were chosen for further experiments.

### Validating lentiviral constructs for SMYD1 over-expression and SMARCD1 suppression in hESC-VCMs

**Figure 3:**
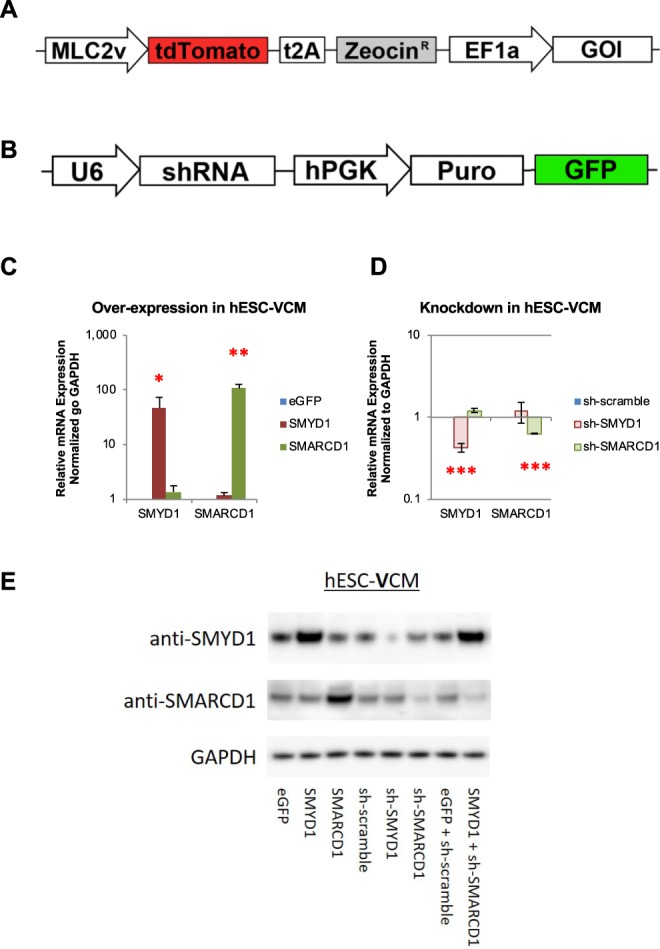
Validation of over-expressions and knockdowns in hESC-VCM. (**A**) Design of the lentiviral constructs to over-express the genes of interest (GOI) – eGFP, SMYD1, or SMARCD1. **(B)** Design of the shRNA lentiviral constructs against the genes of interest (GOI) – sh-scramble, sh-SMYD1, or sh-SMARCD1. The transduction efficiency of the over-expression constructs **(C)** and shRNA knockdown constructs **(D)** in hESC-**V**CM were examined by quantitative real-time PCR. Relative mRNA expression were normalized to GAPDH. S.E.M. of three independent experiments are presented. *p < 0.05; **p < 0.01; ***p < 0.001 (Student’s t-test). **(E)** hESC-**V**CMs were transduced with the over-expression lentiviral constructs and/or shRNA knockdown constructs and protein expressions were validated by Western blotting with either SMYD1 or SMARCD1 antibodies. GAPDH was used to demonstrate equivalent protein loading.

To avoid ambiguities due to presence of non-cardiac cells or other chamber-specific types, a pure ventricular population was isolated by transduction with LV-MLC2v-tdTomato-t2A-zeo, followed by zeocin selection^3^. Lentiviral constructs were also designed to over-express and suppress *SMYD1* and *SMARCD1* in hESC-**V**CM (Fig. 3A,B) so as to mirror their endogenous developmental expression profiles. Over-expression and suppression were confirmed at the transcript and protein levels by quantitative real-time PCR (Fig. 3C,D) and Western blotting (Fig. 3E), respectively. eGFP over-expression and sh-scramble suppression constructs were used as controls. The localization of SMYD1 and SMARCD1 in hESC-**V**CMs transduced with the over-expression constructs were validated by immunofluorescence. While SMYD1 is expressed in both the nucleus and cytosol, the majority of SMARCD1 is expressed in the nucleus (Fig. S1). Furthermore, cell morphology and cell size, as determined by immunostaining as well as cell capacitance (Tables 1 and 2), cell nucleation and elongation, as determined by circularity index, were not different between different genetic manipulations (Fig. S2).

**Table 1:** Action Potential Analysis (by Patch Clamping) – SMYD1.

	Spontaneous		Stimulated at 1 Hz	
	*SMYD1 (n* = *5)*	*sh-SMYD1 (n* = *11)*	*SMYD1 (n* = *5)*	*sh-SMYD1 (n* = *5)*
Amplitude (mV)	71.8 ± 5.3	74.8 ± 4.9	70.6 ± 15.3	98.6 ± 5.5
Upstroke Velocity (mV/ms)	4.6 ± 1.0	29.5 ± 8.5	28.6 ± 9.0	41.8 ± 6.3
Decay Velocity (mV/ms)	−0.32 ± 0.11	−0.55 ± 0.08	−0.46 ± 0.09	−0.62 ± 0.16
APD50 (ms)	470.0 ± 42.6	378.6 ± 61.4	494.5 ± 45.7	327.0 ± 49.4*
APD90 (ms)	609.9 ± 98.2	450.7 ± 71.3	559.2 ± 51.8	391.2 ± 46.6*
MDP (mV)	−48.3 ± 2.2	−53.9 ± 2.1	−55.5 ± 1.6	−56.8 ± 3.6
Membrane Capacitance (pF)	88.2 ± 16.6	83.2 ± 10.6		
Firing Rate (Hz)	0.99 ± 0.16	1.13 ± 0.10

**Table 2:** Action Potential Analysis (by Patch Clamping) – SMARCD1.

	Spontaneous		Stimulated at 1 Hz	
	*SMARCD1 (n* = *5)*	*sh-SMARCD1 (n* = *13)*	*SMARCD1 (n* = *5)*	*sh-SMARCD1 (n* = *4)*
Amplitude (mV)	63.9 ± 4.1	69.5 ± 3.5	95.8 ± 5.0	96.2 ± 7.9
Upstroke Velocity (mV/ms)	2.9 ± 0.7	6.0 ± 1.2	32.2 ± 6.6	30.0 ± 13.4
Decay Velocity (mV/ms)	−0.41 ± 0.08	−0.54 ± 0.08	−0.70 ± 0.15	−1.02 ± 0.36
APD50 (ms)	311.0 ± 52.2	335.7 ± 51.0	381.1 ± 93.8	288.8 ± 64.1
APD90 (ms)	380.7 ± 61.6	433.5 ± 61.1	439.8 ± 98.8	337.5 ± 68.7
MDP (mV)	−47.6 ± 3.3	−51.5 ± 2.4	−59.0 ± 3.5	−59.3 ± 7.3
Membrane Capacitance (pF)	121.7 ± 28.4	89.9 ± 13.9		
Firing Rate (Hz)	1.33 ± 0.15	0.77 ± 0.16		

### SMYD1 over-expression enhanced the Ca^2+^ transient amplitude without altering action potential of single hESC-VCMs

**Figure 4:**
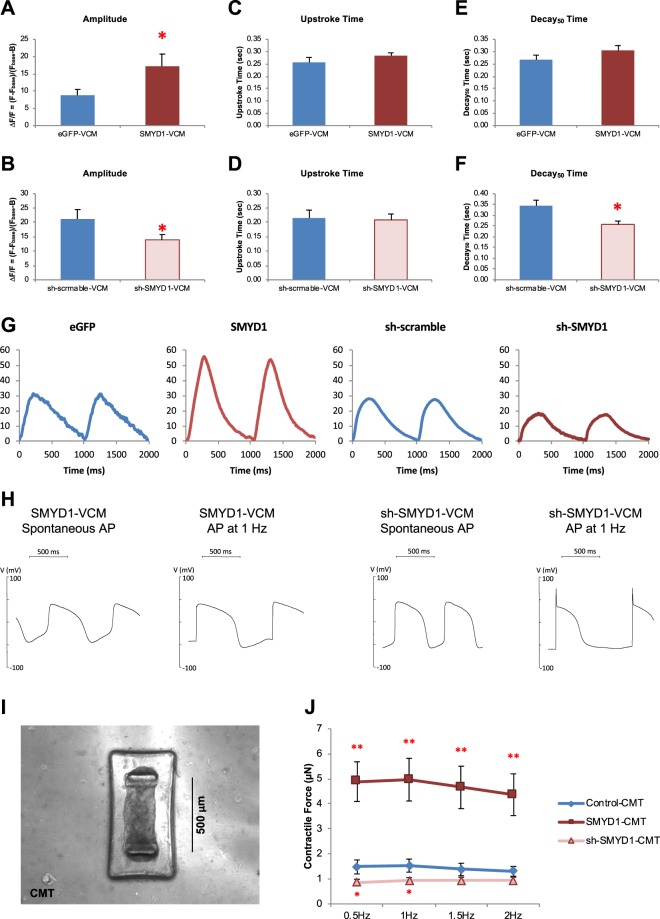
Effects of SMYD1 over-expression. Ca^2+^-transients of eGFP-, SMYD1-, sh-scramble and sh-SMYD1-**V**CMs were measured by calcium imaging. **(A**–**F)** Ca^2+^-transient amplitude, upstroke time and decay_50_ time were calculated. Representative Ca^2+^-transients are shown in **(G)**. Action potentials of eGFP-, SMYD1-, sh-scramble- and sh-SMYD1-**V**CMs were measured with or without electrical stimulation at 1 Hz by patch clamping and representative action potential tracings are shown in **(H)**. **(I)** Each cardiac microtissue (CMT) is composed of 1000 hESC-**V**CMs in a microfabricated mold. The distance between the two cantilevers is 500 µm. **(J)** CMTs were generated from hESC-**V**CMs transduced with (1) eGFP + sh-scramble Control (n = 10), (2) SMYD1 over-expression (n = 14), and (3) sh-SMYD1 suppression (n = 13) lentiviral constructs, and contractile force of the CMTs were measured. *p < 0.05; **p < 0.01 (Student’s t-test vs Control-CMT).

Given the fundamental importance of Ca^2+^ handling and electrophysiology in the biology of hESC-**V**CMs, we first tested for the functional consequences of SMYD1 overexpression and suppression in single hESC-**V**CMs. Figure 4A and B shows that SMYD1-**V**CMs had a significantly larger Ca^2+^ transient amplitude compared to GFP-transduced control (at 1 Hz) (eGFP, n = 17; SMYD1, n = 15; p < 0.05). By contrast, there was a significant decrease in the transient amplitude of sh-SMYD1-**V**CMs compared to sh-scramble control (sh-scramble, n = 13; sh-SMYD1, n = 15; p < 0.05). The upstroke time did not change significantly for both *SMYD1* over-expression and sh-*SMYD1* suppression (Fig. 4C,D). While there was no significant difference in decay_50_ time (time required for 50% of decay) between eGFP-**V**CM and SMYD1-**V**CMs, decay_50_ time was faster in sh-SMYD1 when compared to sh-scramble control (p < 0.05) (Fig. 4E,F). Representative transient tracings are shown in Fig. 4G. The gene expression levels of several Ca^2+^-handling proteins were examined. While SMYD1-**V**CM did not show any statistically significant differences in all the genes tested (n = 6), *CACNA1S* (*Ca*_*v*_*1*.*1*) (p < 0.05), *ITPR3* (*inositol 1*, *4*, *5-triphosphate receptor*, *type 3*) (p < 0.01) and *CACNA1C* (*Ca*_*v*_*1*.*2*) (p < 0.05) were increased in sh-SMYD1-**V**CM (n = 3) (Fig. S3A).

Spontaneously firing and electrically-elicited (1 Hz) action potentials (AP) of single hESC-**V**CMs were measured using the patch-clamp techniques. There were no significant differences in all the parameters of spontaneously firing APs measured between SMYD1- (n = 5) and sh-SMYD1- (n = 11) hESC-**V**CMs (Table 1). When paced at 1 Hz, time for APD_50_ (action potential duration at 50% of repolarization) (p < 0.05) and APD_90_ (action potential duration at 90% of repolarization) (p < 0.05) were significantly shortened in sh-SMYD1-**V**CMs (Table 1), indicating that rate adaptation remained unaltered. Representative AP tracings are shown in Fig. 4H. Transcript levels of potassium (K^+^) and sodium (Na^+^) ion channels were examined by quantitative real-time PCR (Fig, S3B,C). Despite the lack of functional changes, over-expression of *SMYD1* (n = 6) did confer a statistically significant elevation of *KCND3* (*K*_*v*_*4*.*3*) expression (p < 0.01); on the other hand, expression of *KCNA4* (*K*_*v*_*1*.*4*) (p < 0.001), *KCNJ2* (*Kir2*.*1*) (p < 0.05), *SCN3A* (*Na*_*v*_*1*.*3*) (p < 0.01), *SCN4B* (*LQT10*) (p < 0.01) were up-regulated, and *KCNH2* (*hERG*) (p < 0.01) and *SCN5A* (*Na*_*v*_*1*.*5*) (p < 0.001) were down-regulated in sh-SMYD1-**V**CM (n = 3). Such lack of direct correlation between transcript levels and functional expression or consequences has been described previously^22^.

We also examined the expression levels of genes associated with cardiac contractility. *MYH7* (beta-myosin heavy chain, β-MHC) was significantly up-regulated in SMYD1-hESC-**V**CMs (n = 6; p < 0.05); *MYH6* (alpha-myosin heavy chain, α-MHC) was significantly down-regulated in sh-SMYD1-hESC-**V**CM (n = 3; p < 0.001 and p < 0.05) (Fig. S3D). The shift from α- to β-MHC was consistent with maturation of hESC-**V**CMs with increased *SMYD1* expression.

### SMYD1 over-expression increased the contractile force of hESC-VCM-CMTs

We postulated that the improved Ca^2+^ handling after SMYD1 overexpression, along with the shift of α- to β-MHC, would translate into stronger contractile forces as seen in the developmental maturation of cardiomyocytes. To test this notion, we employed a multi-cellular 3D cardiac microtissue (CMT) system developed for measuring dynamic tension^23–25^ (Fig. 4I). This system was superior to conventional measurements of the shortening of single hESC-CMs or their clusters which are merely a surrogate index for contractile forces. CMTs were generated from hESC-**V**CMs transduced with Control, *SMYD1* over-expression and sh-*SMYD1* suppression lentiviral constructs. Over-expressing SMYD1-CMTs exhibited higher maximum contractile forces when compared to GFP control and suppression sh-SMYD1-CMTs with electrical stimulation given at 0.5, 1, 1.5 and 2 Hz (eGFP + sh-scramble Control-CMT, n = 10; SMYD1-CMT, n = 14; sh-SMYD1-CMT, n = 13) (Fig. 4J); at all frequencies tested, sh-SMYD1-CMTs had contractile forces that were only modestly weaker than eGFP + sh-scramble Control-CMT.

### *SMARCD1* suppression increased the gene expression of key Ca^2+^-handling proteins without affecting Ca^2+^-transient and AP properties of hESC-VCMs

**Figure 5:**
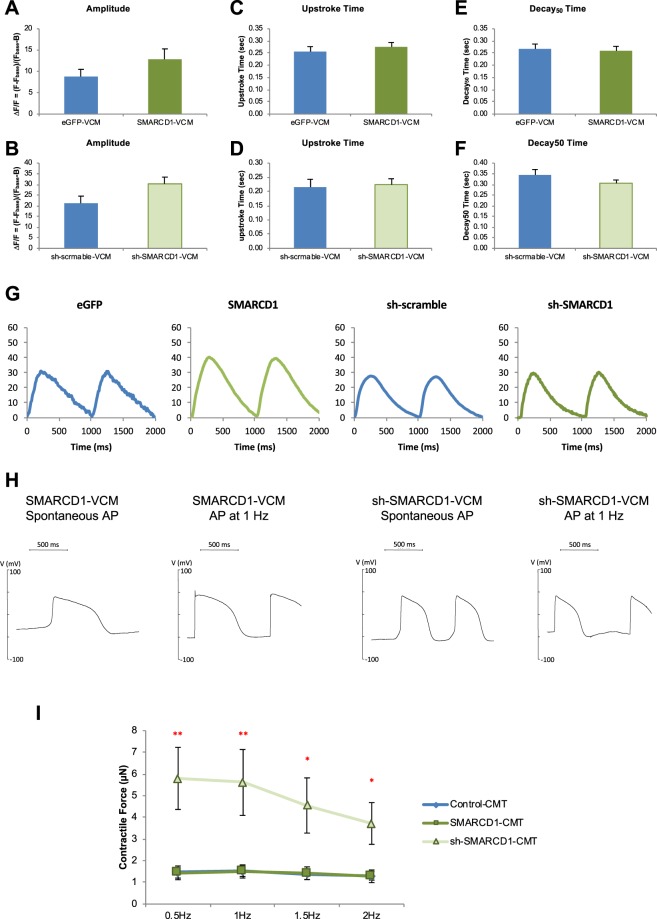
Effects of sh-SMARCD1 suppression. Ca^2+^-transients of eGFP-, SMARCD1-, sh-scramble and sh-SMARCD1-**V**CMs were measured by calcium imaging. **(A**–**F)** Ca^2+^-transient amplitude, upstroke time and decay_50_ time were calculated. Representative Ca^2+^-transient traces are shown in **(G)**. Action potentials of eGFP-, SMYD1-, sh-scramble and sh-SMYD1-**V**CMs were measured with or without electrical stimulation at 1 Hz by patch clamping and representative action potential tracings are shown in **(H)**. **(I)** CMTs were generated from hESC-**V**CMs transduced with (1) eGFP + sh-scramble Control (n = 10), (2) SMARCD1 over-expression (n = 10), and (3) sh-SMARCD1 suppression (n = 8) lentiviral constructs, and contractile force of the CMTs were measured. *p < 0.05; **p < 0.01 (Student’s t-test vs Control-CMT).

Since endogenous *SMARCD1* expression decreased developmentally, we hypothesized that forced-reduction in *SMARCD1* expression would be beneficial to the functionality of hESC-**V**CMs. Ca^2+^-transients and expression of genes encoding for Ca^2+^-handling proteins were examined for SMARCD1 over-expression and sh-SMARCD1 suppression in hESC-**V**CMs. Despite increased mRNA expression of *PLN* (*phospholamban*) and *CASQ2* (*calsequestrin2*) in sh-*SMARCD1*-**V**CMs (p < 0.001 and p < 0.05, respectively) (Fig. S4A), alteration in *SMARCD1* expression levels did not significantly affect Ca^2+^-handling properties (eGFP, n = 17; SMARCD1, n = 11; sh-scramble, n = 13; sh-SMARCD1, n = 14) (Fig. 5A–F). The representative tracings are shown in Fig. 5G. Likewise, electrophysiological profiles of hESC-**V**CMs transduced with SMARCD1 and sh-SMARCD1 lentiviral constructs were characterized. Yet, changes in *SMARCD1* expression did not significantly affect the action potential of hESC-**V**CMs under both spontaneous and electrically stimulated conditions (SMARCD1, n = 5; sh-SMARCD1, n = 13) (Table 2). The representative AP traces are shown in Fig. 5H. The expression levels of several K^+^ and Na^+^ ion channel genes were also examined. While over-expression of *SMARCD1* elevated *KCND3* (*K*_*v*_*4*.*3*) (n = 6; p < 0.01), sh-*SMARCD1* suppression in hESC-**V**CMs (n = 3) significantly increased expression levels of K^+^ channels – *KCNA4* (*K*_*v*_*1*.*4*) (p < 0.05) and *KCNH2* (*hERG*) (p < 0.05), and decreased expression levels of Na^+^ channels SCN5A (p < 0.05) and SCN4B (p < 0.05) (Fig. S4B,C).

### *SMARCD1* suppression promoted contractility in hESC-VCM-CMTs

Despite the lack of effects on Ca^2+^-transients and AP at the single-cell level, CMTs were generated with hESC-**V**CMs transduced with *SMARCD1* constructs for its over-expression and suppression to investigate if any pro- maturational effects could be unleashed at the 3D level. Indeed, sh-*SMARCD1*-CMTs displayed increased maximum contractile forces when electrical stimulation was given at 0.5 to 2 Hz (eGFP + sh-scramble-CMT, n = 10; SMARCD1-CMT, n = 10; sh-SMARCD1-CMT, n = 8) (Fig. 5I). However, there was no significant difference found on the expression levels of genes encoding for cardiac contractile proteins between SMARCD1 over-expressed and sh-SMARCD1 knockdown hESC-**V**CMs (eGFP, n = 6; SMARCD1, n = 6; sh-scramble, n = 3; sh-SMARCD1, n = 3) (Fig. S4D).

### Combined *SMYD1* over-expression and *SMARCD1* suppression synergistically augmented maximum contractile force in hESC-VCM-CMTs and -CTSs

**Figure 6:**
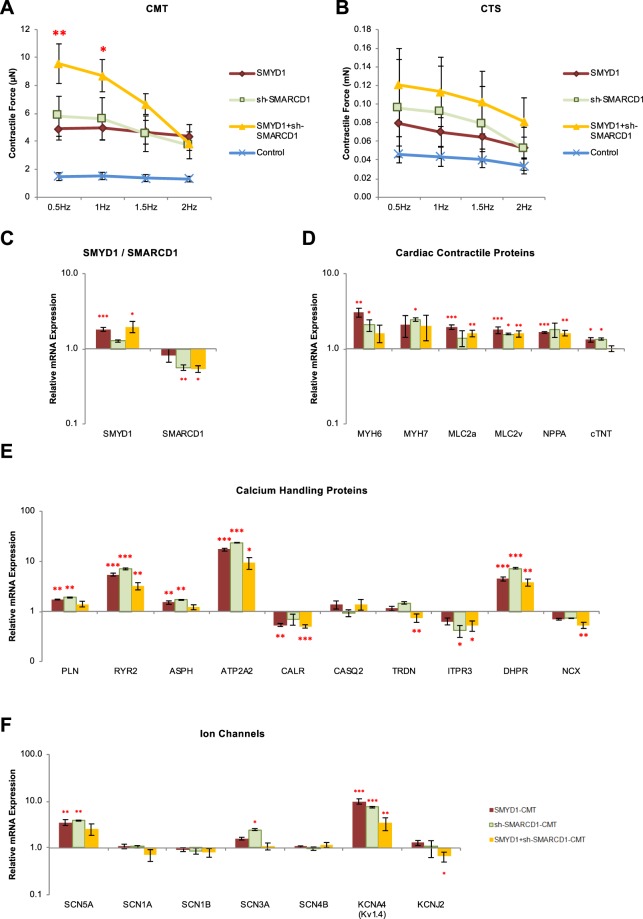
Synergistic effect of SMYD1 over-expression and sh-SMARCD1 suppression. Contractile force of (**A)** CMTs and **(B)** CTSs generated from lentiviral transduced hESC-**V**CMs. CMT: (1) eGFP + sh-scramble Control (n = 10); (2) SMYD1 over-expression (n = 14), (3) sh-SMARCD1 suppression (n = 8), and (4) SMYD + sh-SMARCD1 (n = 11); and CTS: (1) eGFP + sh-scramble Control (n = 10); (2) SMYD1 over-expression (n = 11), (3) sh-SMARCD1 knockdown (n = 6), and (4) SMYD + sh-SMARCD1 (n = 11) *p < 0.05; **p < 0.01 (Student’s t-test). Gene expression analysis of hESC-**V**CM-CMTs. CMTs were collected on Day8 and RNA was extracted for gene expression analysis. **(C)** Validation of SMYD1 over-expression or sh-SMARCD1 knockdown in hESC-**V**CM-CMTs. mRNA expression of genes encoding for **(D)** cardiac contractile proteins, **(E)** Ca^2+^-handling proteins, and **(F)** ion channels were measured by quantitative real-time PCR. S.E.M. of three independent batches are presented. *p < 0.05; **p < 0.01; ***p < 0.001 (Student’s t-test).

To further illustrate and validate the contractile effect of SMYD1 over-expression and SMARCD1 suppression, we performed the same experiments in a larger, more physiological 3D-engineered tissue model, the cardiac tissue strip (CTS), as we recently reported^26^. Unlike the microscales of CMTs, CTSs are approximately one centimeter in length, half a millimeter in diameter, and composed of ~10^6^ cells^26^ (Fig. S5). Qualitatively similar to our CMT results, both SMYD1 over-expression and SMARCD1 suppression led to increased contractile forces of CTS compared to controls (Fig. 6A,B).

Since SMYD1 over-expression and sh-SMARCD1 suppression independently resulted in enhanced contractile forces of both CMTs and CTSs, we next tested if simultaneous SMYD1 over-expression and SMARCD1 suppression would synergistically lead to further enhancements. HESC-**V**CMs were doubly transduced with the same lentiviral constructs followed by forming into CMTs and CTSs over the same period of time. Maximum contractile force was measured with electrical stimulation given at 0.5 Hz to 2 Hz. The contractile forces of the doubly-transduced CMTs were significantly higher than either SMYD1 over-expression or sh-SMARCD1 suppression alone (Fig. 6A) at 0.5 Hz and 1 Hz (eGFP + sh-scramble-CMT, n = 10; SMYD1-CMT, n = 14; sh-SMARCD1-CMT, n = 8; SMYD1 + sh-SMARCD1-CMT, n = 11). Similarly, the doubly-transduced CTSs also showed an increase in contractile force (Fig. 6B) (eGFP + sh-scramble-CTS, n = 10; SMYD1-CTS, n = 11; sh-SMARCD1-CTS, n = 6; SMYD1 + sh-SMARCD1-CTS, n = 11). In addition, doubly-transduced CTSs constructed using h-**V**CMs derived from our in-house control hiPSC^27^ also showed an increased contractile force when compared to control (Fig. S6).

To seek insights into the molecular basis underlying this observation, Control-, SMYD1-, sh-SMARCD1- and SMYD1 + sh-SMARCD1-CMTs were harvested for assessing any transcription changes. Firstly, the expression of *SMYD1* and *SMARCD1* were validated in Fig. 6C, where *SMYD1* was increased in SMYD1-CMTs and SMYD1 + sh-SMARCD1-CMTs, while *SMARCD1* was reduced in sh-SMARCD1-CMTs and SMYD1 + sh-SMARCD1- CMTs. Interestingly, correlating to increased contractile force, there was a 1.5 to 3.0-fold increase in the expression of genes encoding for cardiac contractile proteins in SMYD1-, sh-SMARCD1- and SMYD1 + sh-SMARCD1-CMTs when compared to control (Fig. 6D). Moreover, Ca^2+^-handling proteins – *RYR2* (*ryanodine receptor 2*), *ATP2A2* (*Serca2*), *CACNA1S* (*Ca*_*v*_*1*.*1*) were also significantly up-regulated, as *CALR* (*calreticulin*) and *ITPR3* expressions were reduced (Fig. 6E). The expression of ion channels – *SCN5A* (*Na*_*v*_*1*.*5*) and *KCNA4* (*K*_*v*_*1*.*4*) were increased by 2.5- to 3.8-fold and 3.3 to 9.9-fold, respectively (Fig. 6F).

**Figure 7:**
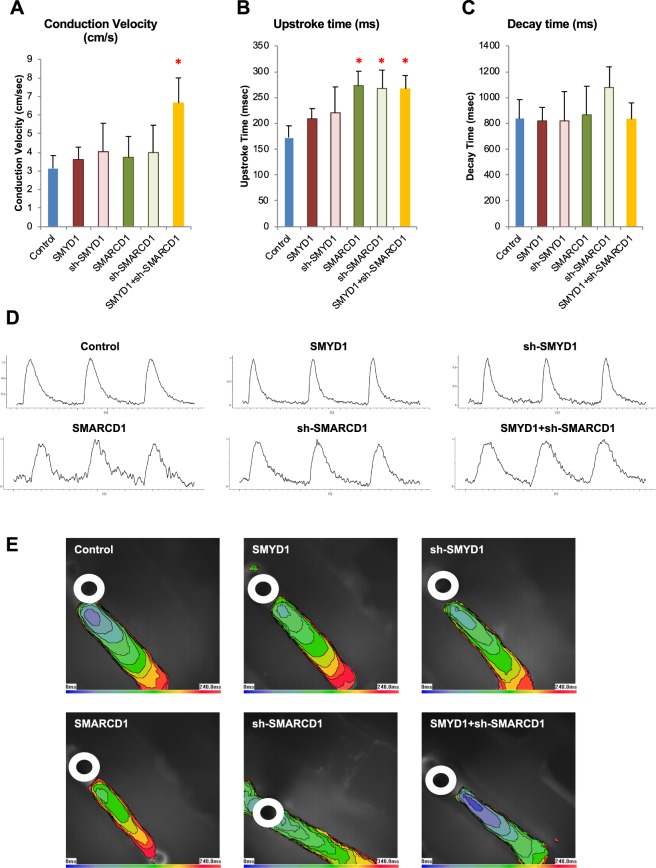
Electrically-induced Ca^2+^-transient profiles in CTS generated from lentivirus transduced hESC-VCMs. (**A)** Conduction velocities, **(B)** average upstroke, and **(C)** average decay time were calculated in CTSs generated from (1) eGFP + sh-scramble Control (n = 7), (2) SMYD1 over-expression (n = 8), sh-SMYD1 suppression (n = 4); (4) SMARCD1 over-expression (n = 4); (5) sh-SMARCD1 suppression (n = 6), and (6) SMYD + sh-SMARCD1 (n = 8) lentiviral transduced hESC-**V**CMs. *p < 0.05 (student t-test vs Control CTS). **(D)** Representative tracings of electrically-induced calcium transient profile in CTS generated from lentivirus transduced hESC-**V**CMs. **(E)** Isochrone map of CTS with 20 ms intervals showing a one-directional spread of optically mapped calcium transient signal away from the origin of the point electrical stimulation (white circle).

For additional functional insights, Ca^2+^-transients were measured by optical mapping in SMYD1 + sh-SMARCD1-CTS. The conduction velocity, as gauged by the speed at which Ca^2+^-transients traveled, was two times higher in the doubly-transduced CTSs when compared to the Control-CTSs (Control-CTS, n = 7; SMYD1-CTS, n = 8; sh-SMYD1-CTS, n = 4; SMARCD1-CTS, n = 4; sh-SMARCD1-CTS, n = 6; SMYD1 + sh-SMARCD1-CTS, n = 8; p < 0.05) (Fig. 7A). While there were no significant differences in the decay times, the upstroke times were increased in SMARCD1-CTS, sh-SMARCD1-CTS and SMYD1 + sh-SMARCD1-CTS (p < 0.05) (Fig. 7B,C). Representative tracings and isochrone maps of electrically-induced calcium transients of CTSs are shown in Fig. 7D,E. Taken together, the results indicated that SMYD1 over-expression and sh-SMARCD suppression sufficed to induce maturation of hESC-**V**CM, CMTs and CTSs. Furthermore, certain effects (e.g., transcriptional changes) that were not detected at the single-cell level could be unleashed under 3D environments.

## Discussion

Epigenetic modulation has been well established as a transcriptional regulatory mechanism ubiquitously involved in development, maintenance, and disease^28^. Genes need to be physically accessible by the transcription machinery in order to be transcribed. Chromatin remodeling proteins confer chemical modifications, including acetylation, methylation and ubiquitination, to histone tails and physically rearrange genomic DNA to activate or repress transcription^29^. Indeed, histone modification enzymes, DNA methyl-transferases and chromatin binding proteins work in concert to remodel the chromatin into open or closed conformations. The temporal and dynamic epigenetic states are conferred by reversible chemical modifications to genomic DNA, typically cytosine, and to residues along the C-terminal tails of histone proteins. Distinct chromatin and gene expression patterns are associated with lineage and cell fate decisions. For instance, genes highly expressed in undifferentiated hESCs lose active H3K4me3 and gain repressive H3K27me3 histone marks over the course of cardiac differentiation; on the contrary, repressive H3K27me3 gradually decreases on genes involved in mesodermal differentiation and cardiac development as active H3K4me3, H3K36me3 and RNA expression appear^3–5^. Despite their importance, chromatin remodeling has not yet been extensively investigated in *in vitro* cardiac differentiation and maturation of hESC/iPSC-**V**CMs.

Using a data-driven approach, we have identified two chromatin remodeling proteins that are differentially expressed in human heart, SMYD1 and SMARCD1, and characterized their roles in the phenotypes of hESC-**V**CMs, CMTs as well as CTSs. In particular, we showed that expression levels of *SMYD1* and *SMARCD1* elicited different responses under 2D or 3D environment. At the single cell level, *SMYD1* over-expression increased the Ca^2+^ transient amplitude but its suppression led to its decrease. Consistent with a positive inotropic effect as a result of the improved Ca^2+^ handling, *SMYD1* over-expression in CMTs also induced increased contractile forces. Furthermore, SMYD1 could also exert its effect on contractile proteins at the genetic level by upregulating myosin genes such as *MYH6* and *MYH7*. Indeed, SMYD1 is very strongly correlated with MYH7b expression, which is the host gene of cardiogenic miR-499. In a high-throughput yeast-two-hybrid screen, SMYD1 was shown to directly bind MYH7b^30^. Additional experiments are required to confirm whether SMYD1 is directly involved in the regulation of such cardiac miRs as miR-499. Unlike *SMYD1* over-expression, however, *SMARCD1* suppression in single hESC-**V**CMs did not significantly promote CM function such as Ca^2+^ transient properties. When Ca^2+^-handling genes were examined, we noticed a significant up-regulation of *PLN* (*phospholamban*), *CASQ2* (*calsequestrin2*) and *TRDN* (*triadin*) gene expression in sh-SMARCD1 transduced hESC-**V**CMs. PLN and TRDN are important inhibitory regulators of ATP2A2 (SERCA2) and cardiac ryanodine receptor (RyR), respectively.

It has been suggested that a 3D culture environment in concert with appropriate temporal and micro-environmental niches would drive maturation of hESC-CMs to a more adult-like state^1,31,32^. Various 3D cell culture model systems have been established recently and have been shown to be pro-maturational^24,26,33–35^. Unlike single hESC-**V**CMs, both *SMYD1* over-expression and *SMARCD1* suppression in the two 3D models, CMT and CTS, independently led to significant pro-maturational effects in the form of increased contractile forces and conduction velocities over the physiological range of electrical pacing. This effect was likewise accompanied by changes in a number of Ca^2+^-handling gene expression, including increased expression of L-type calcium channel (*DHPR*), *ATP2A2* and *RYR2*, as well as their regulatory proteins including *PLN*, *CASQ2*, *ASPH* (*junctin*) and *TRDN*. Of note, these Ca^2+^-handling genes have been shown to be expressed at low levels in hESC-**V**CMs^3^. In addition, modulated expression of *SMYD1* and *SMARCD* in CMT resulted in a decreased expression in *NCX*, which has been shown to be high in hESC-**V**CMs (compared to adult) due to the reliance of these immature CMs on NCX for Ca^2+^-exclusion^36^. Calreticulin (CALR), an embryonic cardiac substitute for CASQ2^37^, was also down-regulated in SMYD1- and sh-SMARCD1-CMTs, consistent with a more mature Ca^2+^-handling phenotype. In addition to Ca^2+^-handling genes, expression of various genes encoding for cardiac contractile proteins including myosin heavy chain –alpha (*MYH6*) and –beta (*MYH7*) as well as myosin light chain -a (*MLC2a*) and –v (*MLC2v*) were also up-regulated, contributing to the increased contractile force. The Na^+^ channel subunit *SCN5A* and the K^+^ channel subunit *KCNA4* are also up-regulated in the CMTs transduced with the three different constructs. Therefore, certain effects that were not detected at the single-cell level could be unleashed under 3D environments. Interestingly, further maturity, as gauged by the improved contractile force and conduction velocity, was attained in CMTs and CTSs co-transduced for simultaneous *SMYD1* over-expression and *SMARCD1* suppression, hinting at a synergistic effect between *SMYD1* and *SMARCD1*. Taken collectively, the overall results were consistent with the notion that epigenetic priming requires additional triggering signals such as 3D non-cell autonomous environmental cues to unleash the effects of these chromatin remodelers for pro-maturation effects.

## Conclusion

Using transcriptomic and bioinformatics analysis as a guide, our functional experiments have revealed that the two chromatin remodelers SMYD1 and SMARCD1 play distinct roles in *in vitro* maturation of hESC-**V**CMs. SMYD1 overexpression and/or SMARCD1 suppression in single hESC-**V**CMs as well as the 3D constructs CMTs and CTSs led to improved contractile as well as electrophysiological phenotypes that signify partial maturation. Some of the effects could be unleashed only under 3D environments, underscoring the importance of non-cell autonomous environmental cues for maturation. Overall, these results were consistent with the notion epigenetic priming requires additional triggering signals for pro-maturation effects^3^.

## Methods

### Cell maintenance, cardiac differentiation and harvest

Undifferentiated human embryonic stem cell line - HES2 (ESI International, Singapore) was maintained at 37 °C with 5% CO_2_ incubator in mTeSR^®^1 culture medium (Stem Cell Technologies). A directed differentiation protocol was used to differentiate HES2 into ventricular cardiomyocytes using the activin A, BMP-4, and IWR1, as reported^27^. The differentiation cultures were maintained in 5% CO_2_/5% O_2_/90% N_2_ environment for the first 8 days, and then transferred into normal 5% CO_2_ incubator. Fresh media is supplied every 3 to 4 days. Human fetal and adult cardiac tissues were isolated and experimented according to protocols approved by UC Davis International Union of Pure and Applied Chemistry (IUPAC) and Institutional Review Board (IRB) after informed consent were obtained from study participants (Protocol #200614787-1 and # 200614594-1). RNA was extracted with Trizol (Invitrogen) following manufacturer protocol.

### Cardiac microtissue preparation

Cardiac microtissues (CMTs) were generated in microfabricated molds as previously described^24^. Briefly, transduced hESC-**V**CMs were passed through a 40 µm strainer (BD Biosciences) and resuspended in Collagen Solution Mix (1X Gibco® Medium-199 (Life Technologies); 10 mM HEPES; 13 mM NaOH; 0.035% w/v NaHCO_3_; 0.5 mg/mL Fibrinogen (Sigma); and 1.5 mg/mL Collagen I (BD Biosciences)) and seeded at 1.2 × 10^6^ per mold. The cells were centrifuged into the microwells at the bottom of the mold and excess Collagen Solution Mix was aspirated. The cells were cultured in Gibco® DMEM medium (Life Technologies) containing 10% FBS and 1% Chicken Embryo Extract (Gemini) for the first 48 hrs, followed by Gibco® DMEM medium (Life Technologies) containing 5% FBS for 6 days. Culture medium was changed daily and compaction of cells was observed within 48 hours after seeding.

### Cardiac tissue strip (CTS) preparation

Cardiac tissue strips (CTS) were generated in bioreactors made of polydimethylsiloxane (PDMS) as previously described^26^. Briefly, 1 × 10^6^ transduced hESC-**V**CMs were resuspended in 100 µL of Collagen-Matrigel Mixture (0.8X MEM (Sigma); 0.6X PBS; 10 mM NaOH; 80 mM HEPES at pH 9; 2 mg/mL Collagen I (Life Techonologies); 0.9 mg/mL Matrigel(Corning)) and added into a rectangular well for each CTS. The cell mixture was then incubated at 37 °C with 5% CO_2_ for 2 hrs to allow the collagen to polymerize. CTS were maintained in high-glucose Dulbecco’s modified Eagle medium (DMEM) containing 10% newborn bovine serum (NCS) (Life Technologies), 1% penicillin/streptomycin (Life Technologies), and 0.2% ampohotericin B (Sigma) with daily half-medium exchanges.

### Gene expression microarrays

The Illumina bead array (Human Ref6 v3.0) was used to assay gene expression of 48,804 probes for each sample. Background subtraction and cubic spline normalization were performed on the array data using the Illumina BeadStudio 3.1 software suite. Annotation of the microarray probes was performed using the lumi package in R and the ReMOAT pipeline^38,39^.

### Bioinformatic analysis

WGCNA was performed on an input matrix of all normalized signal intensity values of 13,372 Illumina microarray probes that mapped to a validated RefSeq gene and were expressed over background signal intensity in at least one sample. The optimal β power coefficient of 9 was calculated to optimize scale free topology. An unsigned adjacency matrix was calculated using the WGCNA R package, using the equation: a(i, j) = |cor(x(i), x(j))|^β^. Dissimilarity is equal to the inverse of adjacency between two genes, or 1 − a(i, j). The modules were assigned by default Dynamic Tree Cut parameters^40^. Dissimilarity values between genes were used to construct hierarchical clustering of the genes and the topological overlap map. Hierarchical clustering was performed on log2 transformed and normalized data using Cluster 3.0, using uncentered correlation and average linkage^41^. Heatmaps were generated with Java TreeView v.1.1.6^42^. Biological process ontological classifications were assigned to groups of genes using the ToppGene suite^43^. P-values were calculated by comparing the number of genes within the test set belonging to a given GO identifier compared with an equivalent number of randomly selected genes from the genome. Bonferroni-corrected p-values < 0.05 were considered to be significant.

### Lentiviral-mediated gene transfer

Cardiospheres were dissociated and re-plated as single cells on Day14 and transduced with recombinant lentivirus particle LV-MLC2v-tdTomato-t2A-Zeocin^R^-EF1α-GOI or U6-shRNA(GOI)-hPGK-Puromycin^R^-t2A-GFP at MOI of 5 on Day15, for over-expression and knockdown, respectively. The genes of interest (GOI) were SMYD1, SMARCD1, and GFP or sh-Scramble as a control. Zeocin^TM^ (300 µg/mL) (Life Technologies) was added to the transduced cells from Day 19 to Day24 to eliminate non-ventricular CMs. The effectiveness using MLC2v-promoter lentivirus for **V**CM selection has been reported previously^36,44^.

### cDNA synthesis and quantitative real-time PCR

cDNA were prepared using QuantiTect Reverse Transcription Kit (Qiagen). Gene expressions were quantified using StepOnePlus^TM^ Real-Time PCR System (Applied Biosystems). PCR amplifications were carried out in 96-well optical plates with 20 µL reaction volume, consisting of 100 ng of cDNA template, 4 pmol of forward and reverse primers, and 1X KAPA SYBR FAST qPCR Master Mix (KAPA Biosystems). The reactions were incubated at 95 °C for 3 min, and followed by 40 to 50 cycles of 95 °C for 3 sec, and 60 °C for 20 sec. Relative mRNA expressions were calculated as fold changes normalized by GAPDH. Primer sequences are listed in Supporting Information Table S1.

### Western blotting

Protein extracts were loaded in 12% NuPAGE Bis-Tris Gel (Life Technologies) and separated by electrophoresis in 1X NuPAGE® MES SDS Running Buffer (Life Technologies) at 100 V for 30 min, followed by 200 V for 2 hrs. The resolved proteins were transferred from the gel onto PVDF membrane in 1X NuPAGE® Transfer Buffer (Life Technologies) containing 20% Methanol at 30 V for 1 hr at room temperature. The PVDF membrane was blocked in 5% instant skim milk prepared in 0.1% v/v PBS + Tween20 (PBST) for 1 hour at room temperature to prevent non-specific binding of antibody. The membrane was probed with SMYD1 (ab32482), SMARCD1 (ab81621) or GAPDH (ab8245) primary antibodies overnight at 4 °C with agitation. After overnight incubation, the membrane was washed three times with PBST to remove excess primary antibody and was then incubated with appropriate secondary antibodies conjugated with horseradish peroxidase (HRP) for 1 hr at room temperature. Finally, the membrane was washed for another three times in PBST and was developed using the ECL Plus Western blotting detection system.

### Immunofluorescence staining of hESC-VCM

Lentiviral transduced hESC-VCMs were seeded in 96-well plate and cultured at 37 °C with 5% CO_2_ in high-glucose Dulbecco’s modified Eagle medium (DMEM) containing 10% fetal bovine serum (Life Technologies). After 48 hrs, hESC-**V**CMs were fixed with freshly prepared 4% paraformaldehyde for 20 min, washed three times with PBS, permeabilized with 0.5% Triton X-100 for 10 min, and washed three times with PBS. The fixed and permeabilized hESC-**V**CMs were then blocked in 1% BSA for 1 hour at room temperature to prevent non-specific binding of antibody. The hESC-**V**CMs were probed with SMYD1 (ab32482), SMARCD1 (ab81621) or cTNT (ab8295) primary antibodies overnight at 4 °C. After overnight incubation, the probed hESC-**V**CMs were washed three times with PBS to remove excess primary antibody and were then incubated with goat anti-rabbit or goat anti-mouse TRITC antibodies (Life Technologies) for 1 hr at room temperature. Finally, the stained hESC-VCMs were washed for another three times in PBS and ProLong® Gold Antifade Mountant with DAPI (Life Technologies) was added for the staining of nucleus and protection from photobleaching. Immunofluorescence images were taken with 20X objective using Nikon DS-Qi2 camera.

### Calcium imaging of hESC-VCM

The intracellular Ca^2+^ ([Ca^2+^]_i_) transients were analyzed by loading the cells with 1.5 μM X-Rhod-1 (Invitrogen, Carlsbad, CA) for 10 minutes at 37 °C in Tyrode’s solution containing: 140 mM NaCl, 5 mM KCl, 1 mM MgCl_2_, 1 mM CaCl_2_, 10 mM HEPES and 10 D-glucose at pH 7.4, followed by imaging with a spinning disc laser confocal microscope (PerkinElmer). The electrically-induced Ca^2+^ transients (E[Ca^2+^]_i_) were triggered by pulses (40 ms pulse duration; 40 V/cm; 1 Hz) generated from a field generator. The amplitudes of Ca^2+^-transients are presented as the background corrected pseudoratio (ΔF/F)^1,2^ = (F − F_base_)/(F_base_ − B) where F_base_ and F is the measured fluorescence intensity before and after stimulation, respectively, and B is the average background signal from areas adjacent to the targeted cell. The transients rise (V_upstroke_) and the transients decay (V_decay_) were subsequently calculated and analyzed.

### Characterization of electrophysiological function

For electrical recording, the whole-cell conFigureuration of the patch-clamp technique was used with an EPC-10 amplifier and Pulse software (Heka Elektronik, Lambrecht, Germany). Patch pipettes were prepared from 1.5 mm thin-walled borosilicate glass tubes using a Sutter micropipette puller P-97 and had typical resistances of 3-5MΩ when filled with an internal solution containing: 110 mM K^+^ aspartate, 20 mM KCl, 1 mM MgCl_2_, 0.1 mM Na-GTP, 5 mM Mg-ATP, 5 mM Na_2_-phospocreatine, 1 mM EGTA, 10 mM HEPES, pH adjusted to 7.3 with KOH. The external Tyrode’s bath solution consisted of: 140 mM NaCl, 5 mM KCl, 1 mM CaCl_2_, 1 mM MgCl_2_, 10 mM glucose, 10 mM HEPES, pH adjusted to 7.4 with NaOH. The tip potential was zeroed before the patch pipette contacted the cell. Upon seal formation and followed by patch break, the capacitance compensation was applied. Series resistance compensation was used up to 80%. Action potentials were recorded with the current-clamp mode. Experiments were performed at 37°C.

### Optical mapping of CTS

CTSs were incubated with the calcium indicator X-Rhod-1 AM at 5 μM, (Life Technologies) for 45 min at 37 °C in NCS media. The staining solution was then removed and replaced by Tyrode’s solution consisting of 140 mM NaCl, 5 mM KCl, 1 mM MgCl2, 1 mM CaCl2, 10 mM glucose, and 10 mM HEPES at pH 7.4. A MiCAM Ultima-L Dual Camera System (SciMedia, USA), was used to measure changes in dye fluorescence. The system consists of 1x objective and 1x condenser with a high-speed CMOS camera which gives a field of view of 10 mm × 10 mm at a resolution of 100 × 100 pixels. Imaging was performed at 5-sec intervals. Excitation of the dye was completed using a halogen light source (HL-151, Moritex Schott, Japan) mounted with emissions filters. Point electrical stimulations at different frequencies were applied at one end of the CTS at 10 V and 10 ms duration. Data collection and analysis were completed with BrainVision software (SciMedia, USA). Parameters including upstroke time, decay time and conduction velocity were calculated as previously described^45^.

### Force measurements

CMTs within the microfabricated molds were imaged using Prosillica GX camera (Allied Vision). Movement of the cantilevers was tracked using SpotTracker plugin in ImageJ (National Institute of Health). The spring constant of the cantilevers was calculated empirically and was used to transform deformation of the cantilevers into μN of force^5^. For CTS, the deflections of the posts were captured in real time with a high-speed camera (100 frames/s) and LabView software (National Instruments, USA). The twitch force (mN) was calculated by applying a beam-bending equation from elasticity theory^26^.

The datasets generated during and/or analyzed during the current study are available from the corresponding author on reasonable request.

## Supplementary information

Supporting information
