# Supplementary material for: Modulation of chromatin remodeling proteins SMYD1 and SMARCD1 promotes contractile function of human pluripotent stem cell-derived ventricular cardiomyocyte in 3D-engineered cardiac tissues
Source: Sci Rep. 2019 May 16;9:7502. doi: 10.1038/s41598-019-42953-w (PMC6522495; doi:10.1038/s41598-019-42953-w)
Supplement: Supplementary file 1 — Supporting information [file 41598_2019_42953_MOESM1_ESM.pdf]

Supplementary Information

Manuscript title:

Modulation of chromatin remodeling proteins SMYD1 and SMARCD1 promotes contractile function of human pluripotent stem cell-derived ventricular cardiomyocyte in 3D-engineered cardiac tissues.

Author List:

Maggie Zi-Ying Chow, Stephanie N. Sadrian, Wendy Keung, Lin Geng, Lihuan Ren, Chi-Wing Kong, Andy On-Tik Wong, Jean-Sebastien Hulot, Christopher S. Chen, Kevin D. Costa, Roger J Hajjar, Ronald A. Li*.

*Corresponding Author

Email: ronald.li@ki.se

S1 Fig

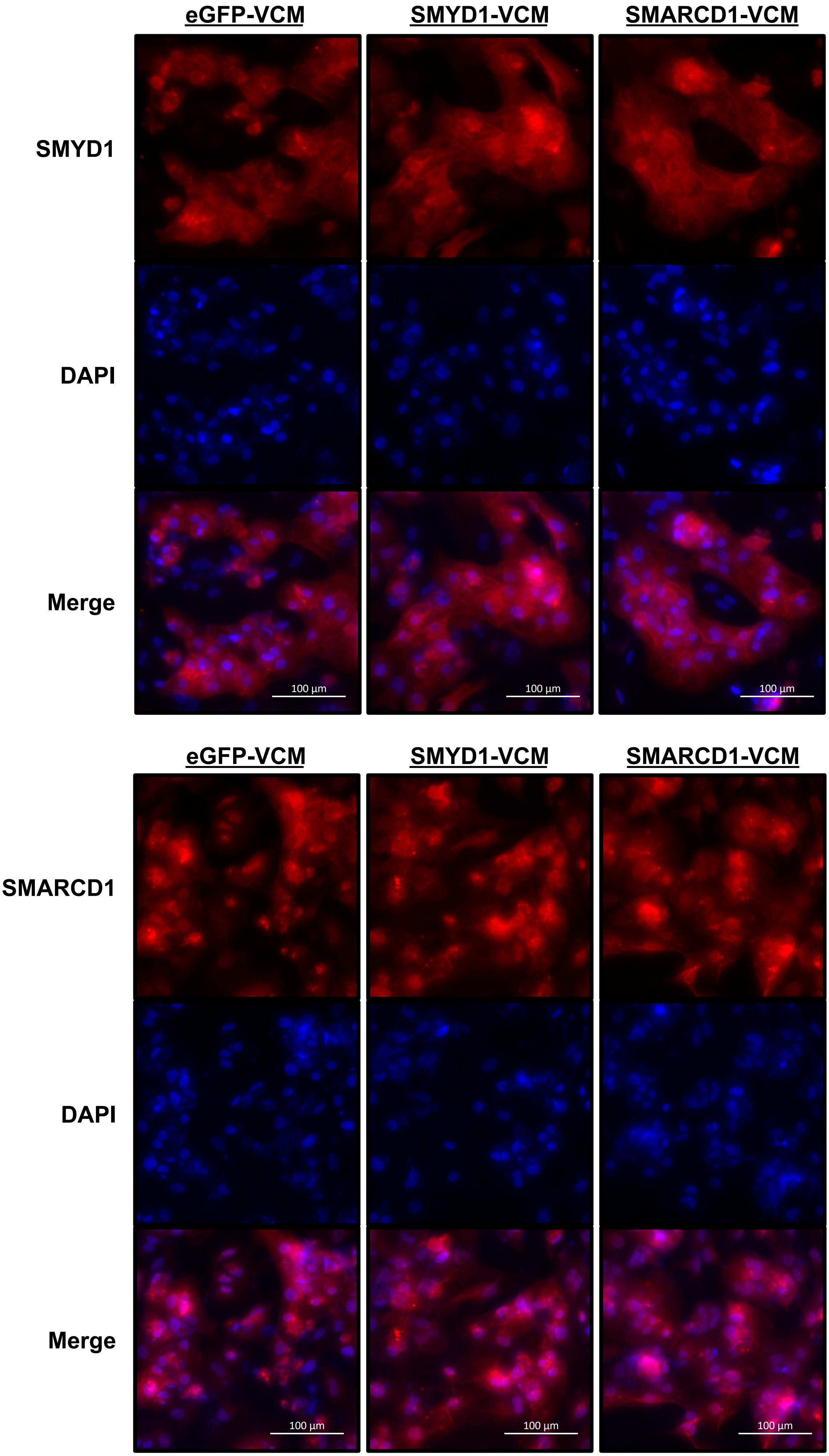
eGFP-VCM
SMYD1-VCM
SMARCD1-VCM
SMYD1
DAPI
Merge
100 µm
100 µm
100 µm
eGFP-VCM
SMYD1-VCM
SMARCD1-VCM
SMARCD1
DAPI
Merge
100 µm
100 µm
100 µm

Fig S1 **Immunofluorescence staining of SMYD1 and SMARCD1 in transduced hESC-VCM.** hESC-**V**CMs were transduced with eGFP, SMYD1 or SMARCD1 lentiviral over-expression constructs and immuno-stained with antibodies against SMYD1 (red, top panel) and SMARCD1 (red, bottom panel). Scale bar = 100 μM.

S2 Fig

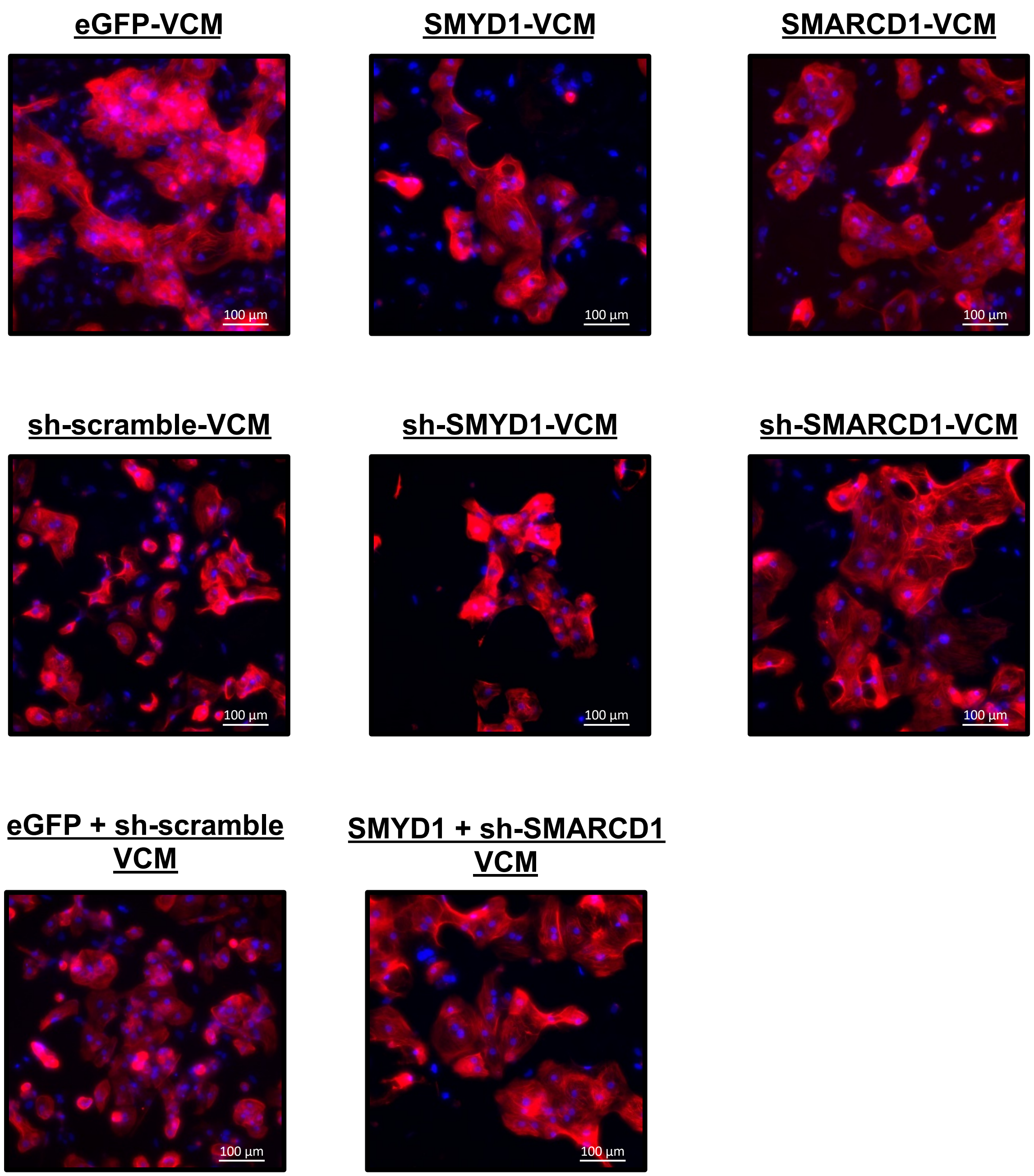
eGFP-VCM
SMYD1-VCM
SMARCD1-VCM
sh-scramble-VCM
sh-SMYD1-VCM
sh-SMARCD1-VCM
eGFP + sh-scramble VCM
SMYD1 + sh-SMARCD1 VCM
100 µm

Fig S2 **Representative immunofluorescence staining of cardiac troponin T (cTNT) in lentiviral transduced hESC-VCMs.** hESC-**V**CMs were transduced with eGFP, SMYD1 or SMARCD1 lentiviral over-expression constructs and/or sh-scramble, sh-SMYD1 or sh-SMARCD1 lentiviral knockdown constructs and immuno-stained with antibodies against cTNT (red). Scale bar = 100 μM.

S3 Fig

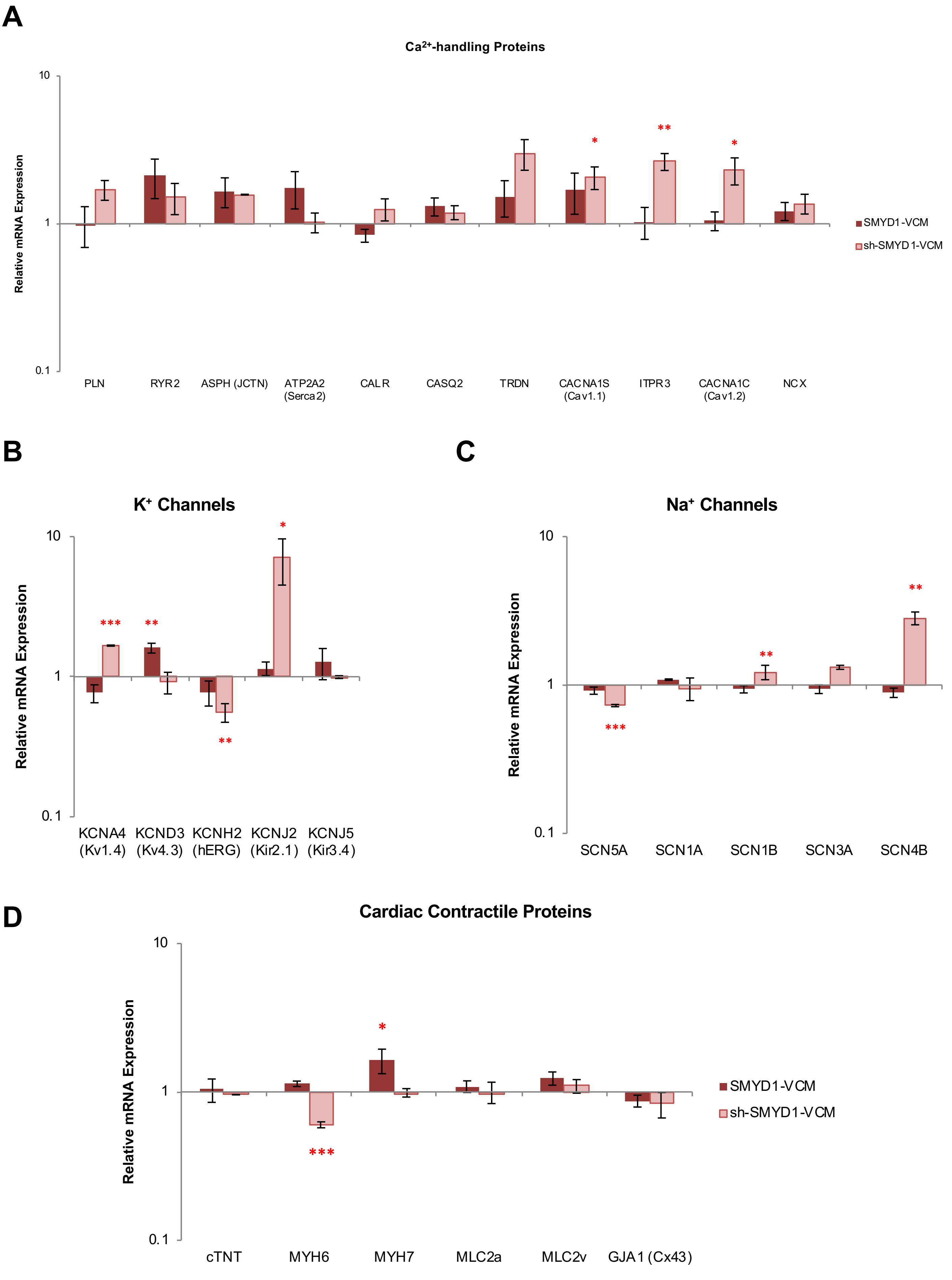
A
Ca²⁺-handling Proteins
Relative mRNA Expression
10
1
0.1
PLN
RYR2
ASPH (JCTN)
ATP2A2 (Serca2)
CALR
CASQ2
TRDN
CACNA1S (Cav1.1)
ITPR3
CACNA1C (Cav1.2)
NCX
SMYD1-VCM
sh-SMYD1-VCM
B
K⁺ Channels
Relative mRNA Expression
KCNA4 (Kv1.4)
KCND3 (Kv4.3)
KCNH2 (hERG)
KCNJ2 (Kir2.1)
KCNJ5 (Kir3.4)
C
Na⁺ Channels
Relative mRNA Expression
SCN5A
SCN1A
SCN1B
SCN3A
SCN4B
D
Cardiac Contractile Proteins
Relative mRNA Expression
cTNT
MYH6
MYH7
MLC2a
MLC2v
GJA1 (Cx43)
SMYD1-VCM
sh-SMYD1-VCM

Fig S3 **Gene expression analysis of SMYD1 over-expression and suppression.** The levels of mRNA transcripts of **(A)** Ca^{2+}-handling proteins, **(B)** K^+ ion channels, **(C)** Na^+ ion channels, and **(D)** cardiac contractile proteins in SMYD1-, and sh-SMYD1-**V**CMs were compared using quantitative real-time PCR. Fold changes of mRNA expression shown are relative to eGFP- and sh-scramble-**V**CMs Control, respectively. GAPDH expression was used for normalization. S.E.M. of at least three independent batches are presented. * $p < 0.05$; ** $p < 0.01$; *** $p < 0.001$ (Student's t-test).

S4 Fig

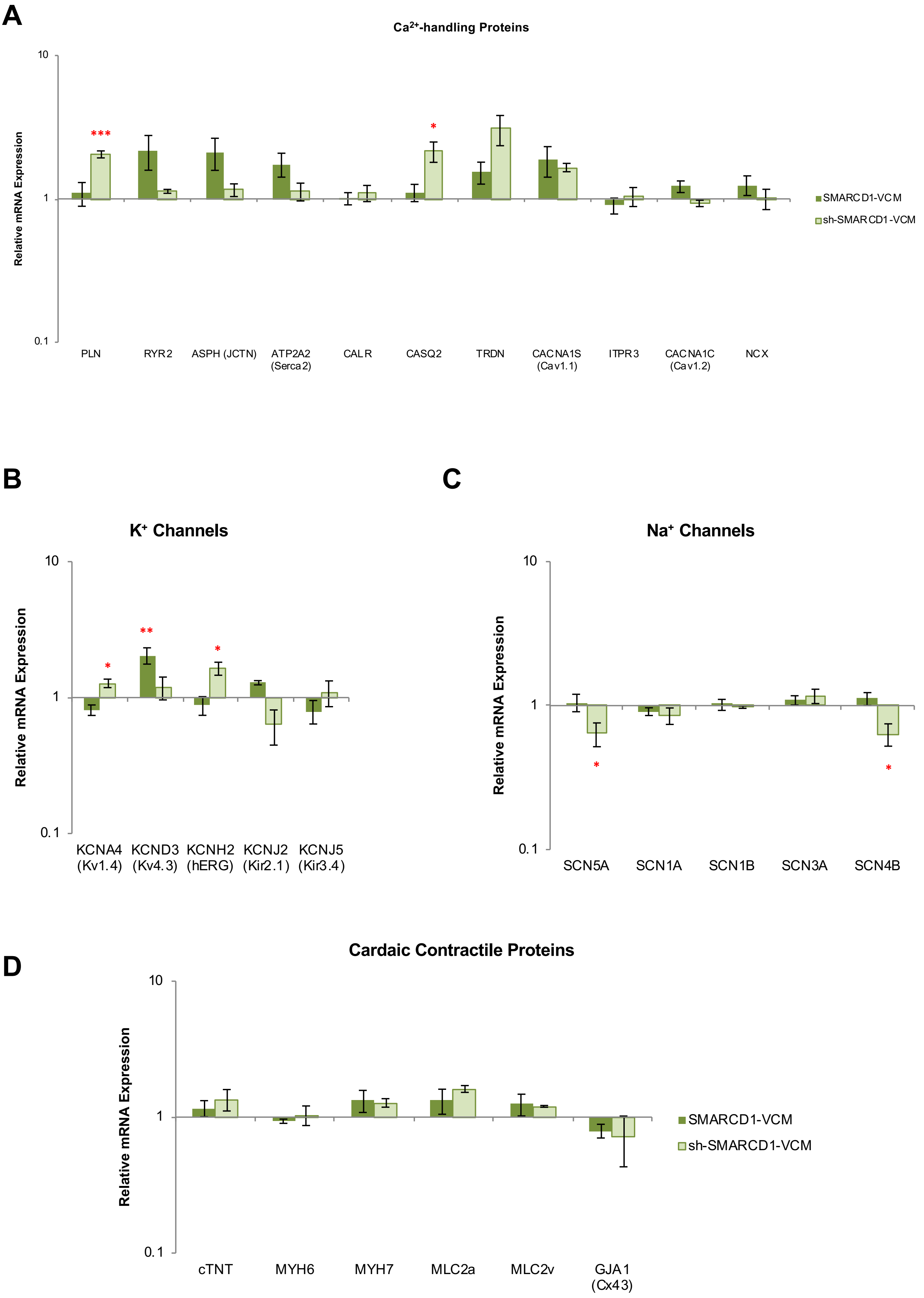

A
Ca2+-handling Proteins
Relative mRNA Expression
10
1
0.1
***
*
PLN
RYR2
ASPH (JCTN)
ATP2A2 (Serca2)
CALR
CASQ2
TRDN
CACNA1S (Cav1.1)
ITPR3
CACNA1C (Cav1.2)
NCX
SMARCD1-VCM
sh-SMARCD1-VCM
B
K+ Channels
Relative mRNA Expression
10
1
0.1
*
**
*
KCNA4 (Kv1.4)
KCND3 (Kv4.3)
KCNH2 (hERG)
KCNJ2 (Kir2.1)
KCNJ5 (Kir3.4)
C
Na+ Channels
Relative mRNA Expression
10
1
0.1
*
*
SCN5A
SCN1A
SCN1B
SCN3A
SCN4B
D
Cardaic Contractile Proteins
Relative mRNA Expression
10
1
0.1
cTNT
MYH6
MYH7
MLC2a
MLC2v
GJA1 (Cx43)
SMARCD1-VCM
sh-SMARCD1-VCM

Fig S4 **Gene expression analysis of SMARCD1 over-expression and suppression.** The levels of mRNA transcripts of **(A)** Ca^{2+}-handling proteins, **(B)** K^+ ion channels, **(C)** Na^+ ion channels, and **(D)** cardiac contractile proteins in SMARCD1-, and sh-SMARCD1-**V**CMs were compared using quantitative real-time PCR. Fold changes of mRNA expression shown are relative to eGFP- and sh-scramble-**V**CMs Control, respectively. GAPDH expression was used for normalization. S.E.M. of at least three independent batches are presented. * $p < 0.05$; ** $p < 0.01$; *** $p < 0.001$ (Student's t-test).

S5 Fig

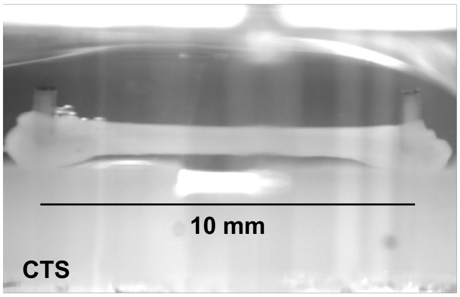

Fig S5 **Representative picture of 3D-Cardiac tissue strip (CTS).** Each CTS is composed of 1 x 10^6 hESC-**V**CMs in a PDMS mold. The distance between the two posts is 10 mm.

S6 Fig

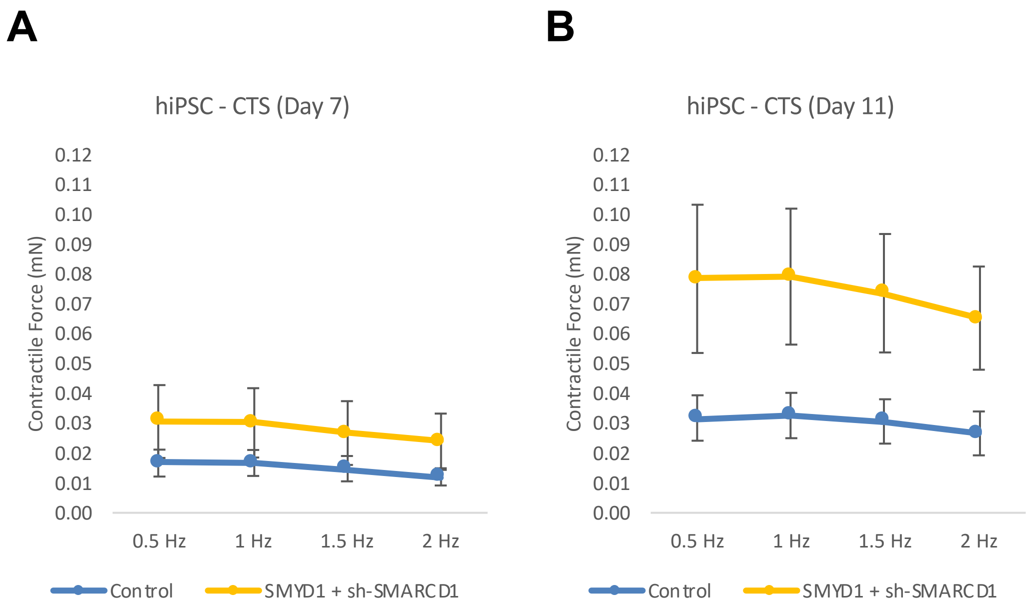
A
B
hiPSC - CTS (Day 7)
hiPSC - CTS (Day 11)
Contractile Force (mN)
0.12
0.11
0.10
0.09
0.08
0.07
0.06
0.05
0.04
0.03
0.02
0.01
0.00
0.5 Hz
1 Hz
1.5 Hz
2 Hz
Control
SMYD1 + sh-SMARCD1

Fig S6 **Synergistic effect of SMYD1 over-expression and sh-SMARCD1 suppression in hiPSC-CTS.**

Contractile force of CTSs made by lentiviral transduced hiPSC-**V**CMs. (1) eGFP+sh-scramble Control (n = 4); (2) SMYD+sh-SMARCD1 (n = 3) on **(A)** Day7 and **(B)** Day11 after CTS generation.

Table S1. Primer sequences and shRNA target sequences.

cDNA primers

SMYD1 F'	TGGCCCAACTGTACTGTCAT
SMYD1 R'	CCGGAGCTCAATTCTCATCT
SMARCD1 F'	ATGACACCTCAGGGACCTTC
SMARCD1 R'	GGACTGATCCATCCCTGACT
MYH6 F'	CAGCACAGAGCTCTTCAAGC
MYH6 R'	GTCCGAGATTTCCTCCTGAA
MYH7 F'	GAGACTGTCGTGGGCTTGTA
MYH7 R'	CTTCTCAATAGGCGCATCAG
MLC2a F'	CAGGCCCAACGTGGTTCTT
MLC2a R'	CCATCACGATTCTGGTCGATAC
MLC2v F'	CCTTGGGCGAGTGAACGT
MLC2v R'	GGGTCCGCTCCCTTAAGTTT
NPPA F'	ATGAGCTCCTTCTCCACCAC
NPPA R'	TCCAGCAAATTCTTGAAATCC
CTNT F'	AAGAGGCAGACTGAGCGGGAAA
CTNT R'	AGATGCTCTGCCACAGCTCCTT
PLN F'	AGCTGCCAAGGCTACCTAAA
PLN R'	GCTGAGCGAGTGAGGTATTG
RYR2 F'	TGGTCCGGTTAGAGATGACA
RYR2 R'	TTGTAATTGCGCTCCTGTTC
ASPH F'	GATGACTCCTTTGAGCACGA
ASPH R'	AGAGTTTCCCAAGCTTGCAT
ATP2A2 F'	TGTAGCAGCCATTCCTGAAG
ATP2A2 R'	AACCAAGGGTTTCCACAGAC
CALR F'	ACCTCTGGCAGGTCAAGTCT
CALR R'	GTCTCGTTGCCAAACTCCTC
CASQ2 F'	ATGGCTACGAATTCCTGGAG
CASQ2 R'	AACGAGCAGAGGAAAGTCGT
TRDN F'	CAGCTGTGTCAAAGCATGAA
TRDN R'	GTTCTGTCGGTAAGGGAGGT
ITPR3 F'	CAGCTCTCCAGGCACAATAA
ITPR3 R'	AGGCTGAGCATGGAAGAGAT
DHPR F'	CAAGTTTGACTTTGACAATGTTCTG
DHPR R'	TGATGTAGATGATGAAGAAGATGGA
NCX F'	GCTTGGTGGCTTCACAATAA
NCX R'	TTCCTCTTTGCTGGTCAGTG
SCN5A F'	AGAGGAGTCTCGCCACAAGT
SCN5A R'	GACCACCAACTTCACTCCCT
SCN1A F'	TGGATAGAGACCATGTGGGA
SCN1A R'	GCAAGGCCAGAAAGAGATTC
SCN1B F'	GTCTACCGCCTGCTCTTCTT
SCN1B R'	TGTCTCTGTTGGCTTTGTCC
SCN3A F'	CTACTCAGAACTCGGTGCCA
SCN3A R'	TTGCTCCAACAAGAGCATTC
SCN4B F'	CGGGAGAAGAAGAAGGAGTG
SCN4B R'	GAAGGTGGTTTCTCCTCTGC
KCNA4 F'	GAGTTTAGGGACGACAGGGA
KCNA4 R'	GTGCCCTGAGTTCTCCAGAT
KCNJ2 F'	CACTTGGTGGAAGCTCATGT
KCNJ2 R'	ACGATCGATTCCACTGTCAA
GAPDH F'	GAAATCCCATCACCATCTTCCAGG
GAPDH R'	GAGCCCCAGCCTTCTCCATG
HPRT F'	TGACACTGGCAAAACAATGCA
HPRT R'	GGTCCTTTTCACCAGCAAGCT
PCNA F'	GGTGTTGGAGGCACTCAAGG
PCNA R'	CCAAAGAGACGTGGGACGAG

shRNA target sequences

sh-SMYD1
CCGGCGCACATCTTCGGAGTGATTACTCGAGTAATCACTCCGAAGATGTGCGTTTTTT
sh-SMARCD1
CCGGGAGACGTGAATGTACGGTGTACTCGAGTACACCGTACATTCACGTCTCTTTTTT
sh-scramble
CCGGGAGCTAATGAACGATAAACTTTCTCGAGAAAGTTTATCGTTCATTAGCTCTTTTT
